# Similarities and Differences in Barriers and Opportunities Affecting Climate Change Adaptation Action in Four North American Landscapes

**DOI:** 10.1007/s00267-017-0933-1

**Published:** 2017-09-07

**Authors:** Whitney R. Lonsdale, Heidi E. Kretser, Cheryl-Lesley B. Chetkiewicz, Molly S. Cross

**Affiliations:** 1 Cornell University, 502N 9th Ave, Bozeman, MT 59715 USA; 2 Wildlife Conservation Society & Cornell University, Saranac Lake, NY 12983 USA; 3 Wildlife Conservation Society Canada, Thunder Bay, ON P7B 6T8 Canada; 4 Wildlife Conservation Society, Bozeman, MT 59715 USA

**Keywords:** Adaptation, Adirondacks, Alaska, Climate change, Ontario, Rocky mountains

## Abstract

Climate change presents a complex set of challenges for natural resource managers across North America. Despite recognition that climate change poses serious threats to species, ecosystems, and human communities, implementation of adaptation measures is not yet happening on a broad scale. Among different regions, a range of climate change trajectories, varying political contexts, and diverse social and ecological systems generate a myriad of factors that can affect progress on climate change adaptation implementation. In order to understand the general versus site-specific nature of barriers and opportunities influencing implementation, we surveyed and interviewed practitioners, decision-makers, and scientists involved in natural resource management in four different North American regions, northern Ontario (Canada), the Adirondack State Park (US), Arctic Alaska (US), and the Transboundary Rocky Mountains (US and Canada). Common barriers among regions related to a lack of political support and financial resources, as well as challenges related to translating complex and interacting effects of climate change into management actions. Opportunities shared among regions related to collaboration, funding, and the presence of strong leadership. These commonalities indicate the importance of cross-site learning about ways to leverage opportunities and address adaptation barriers; however, regional variations also suggest that adaptation efforts will need to be tailored to fit specific ecological, political, social and economic contexts. Comparative findings on the similarities and differences in barriers and opportunities, as well as rankings of barriers and opportunities by region, offers important contextual insights into how to further refine efforts to advance adaptation actions in those regions.

## Introduction

As the climate changes, scientists, policy makers, municipal planners, and natural resource managers face the challenge of assessing impacts and planning adaptation actions to manage the already complex systems for which they are responsible. While adaptation planning within social and ecological systems is progressing (NRC 2010; Bierbaum et al. 2013), implementation of adaptation actions is not keeping pace with an ever-increasing need (Archie et al. 2012; Runhaar et al. 2012; Bierbaum et al. 2013; Kemp et al. 2015). To understand this “stubborn gap” between the perception of climate change as a significant issue and the development of on-the-ground adaptation actions (Betsill and Bulkeley 2007: 448), a growing body of research addresses the barriers and opportunities affecting adaptation (Bassett and Shandas 2010; Uittenbroek et al. 2012; Lemieux et al. 2013; Kemp et al. 2015). Generally, barriers are considered factors that may hamper the process of developing and implementing climate change adaptation actions, but can be overcome using a variety of means and approaches (Moser and Ekstrom 2010); opportunities (also referred to as drivers, bridges, triggers, stimuli) are factors that can promote, enable, or strengthen adaptation planning and implementation (West et al. 2009; Jantarasami et al. 2010; Engle 2012).

Research on factors that impede adaptation actions has taken place at an international scale across a variety of sectors (Measham et al. 2011; Dannevig et al. 2012; Broto and Bulkeley 2013; Aylett 2015). While certain sectors, locales, or political frameworks may produce unique challenges for planners, managers and decision-makers working to address climate change, a core of common barriers seems to exist, including lack of resources (Lemieux et al. 2015; Aylett 2015; Nordgren et al. 2016), lack of information (Aylett 2015; Kemp et al. 2015; Runhaar et al. 2012), lack of leadership (Flugman et al. 2011; Hamin et al. 2014), and competing priorities (Measham et al. 2011; Ellenwood et al. 2012). Within each of these general barrier categories, more specific factors emerge. A lack of funding is often cited as the top resource-related barrier, while insufficient staff and lack of time also reduce the capacity to adapt to climate change (West et al. 2009; Flugman et al. 2011; Bierbaum et al. 2013). Information-related barriers include lack of information at scales relevant to management (Jantarasami et al. 2010; Archie et al. 2012; Kemp et al. 2015), difficulty understanding climate science (Nordgren et al. 2016), and the challenge of transforming knowledge into action (Measham et al. 2011; Aylett 2015). Lack of leadership within organizations and agencies charged with adapting to climate change is a frequently cited barrier, while a lack of political will among government officials has also been noted (Hamin et al. 2014; Aylett 2015). The barrier of competing priorities may be a reflection of resource deficits, but may also indicate conflicting values, within an organization or at the community or governmental level (Aylett 2015; Shi et al. 2015).

Despite the significant range and depth of factors impeding efforts to address climate change, research shows that planning and implementation of adaptation actions are taking place in many countries around the world (Aylett 2015). Where such progress is being made, available funding (Lemieux et al. 2015; Shi et al. 2015) and political and organizational leadership (Burch 2010; Measham et al. 2011; Nordgren et al. 2016) are commonly cited as key drivers, highlighting the fact that certain factors can either enable or impede adaptation. Other frequently cited factors enabling climate change adaptation include awareness of present or future climate change impacts (Tompkins et al. 2010; Shi et al. 2015) and collaboration and sharing of information between organizations and agencies (Juhola and Westerhoff 2011; Lemieux et al. 2015; Nordgren et al. 2016).

The incorporation of adaptation into everyday action and decision-making within the natural resource management and conservation sector, specifically, has also been slow to emerge, despite the fact that many natural resource management agencies have high-level mandates to include climate change in policies and planning (Ellenwood et al. 2012; Kemp et al. 2015). Natural resource managers face many of the common barriers and opportunities discussed above, with lack of financial and human resources cited almost universally, especially by managers in state and federal agencies (Jantarasami et al. 2010; Archie et al. 2012; Kemp et al. 2015). Despite these commonalities, there are some specific factors affecting adaptation in the natural resource management and conservation sector. Upper-level organizational directives may be unclear, leaving managers uncertain about what to prioritize or how to translate climate science into management initiatives (Archie et al. 2012). Ecosystems and species of concern for natural resource managers often span a mosaic of land ownership jurisdictions, adding complexity to the process of planning and implementing adaptation actions (Jantarasami et al. 2010; Lemieux et al. 2015). As public land is often managed for multiple uses, reconciling competing and potentially incompatible land uses make implementing climate adaption actions challenging (Ellenwood et al. 2012). Additionally, as federal, state and provincial land management decisions must include public consultation, natural resource managers cite insufficient stakeholder support or public opposition as hurdles to implementation (Jantarasami et al. 2010; Archie et al. 2012). Managers have also noted that the extensive scope of responsibility and organizational size—of federal agencies in particular—can slow the integration of climate change science into management policies (Archie et al. 2012).

Although a small number of studies do focus on the policies, processes, and organizations/agencies responsible for natural resource management and climate change adaptation in rural and wild landscapes (e.g., Jantarasami et al. 2010; Archie et al. 2012; Ellenwood et al. 2012; Kemp et al. 2015; Lemieux et al. 2015), research on adaptation barriers and opportunities has largely focused on urban planning (e.g., Preston et al. 2010; Flugman et al. 2011; Uittenbroek et al. 2012), and coastal zone and water management (Biesbroek et al. 2013). Additionally, research has most often involved single or neighboring regions and municipalities (Burch 2010; Dannevig et al. 2012; Broto and Bulkeley 2013), leaving a need for comparative studies across contexts, particularly in addressing biodiversity conservation (Biesbroek et al. 2013; Eisenack et al. 2014). Recent studies also identify the need to move beyond basic enumeration of barriers into a more nuanced understanding of the factors both within a particular context and across contexts to understand how they might be addressed (Biesbroek et al. 2013). Research suggests that the “counterpoint” to a particular barrier may not be the most effective way to overcome it (Biesbroek et al. 2013: 1125); for example, if a lack of human resources is cited as a barrier, simply hiring more staff may not help if the leadership and political will are not present to ensure that new staff are incorporating climate change into their work. Therefore, investigation of opportunities in addition to barriers adds important insight into what is actually driving climate change adaptation actions and how specific barriers might be overcome (Eisenack et al. 2014). Research, particularly qualitative inquiry, focusing on perceptions and experiences of key individuals in resource management working on adaptation is recognized as a valuable tool for understanding what is limiting adaptation and how efforts can best be advanced (Tompkins et al. 2010). In summary, there is a recognized need for in-depth, cross-site investigation of factors inhibiting and promoting climate change adaptation, particularly in the field of natural resource management.

**Fig. 1:**
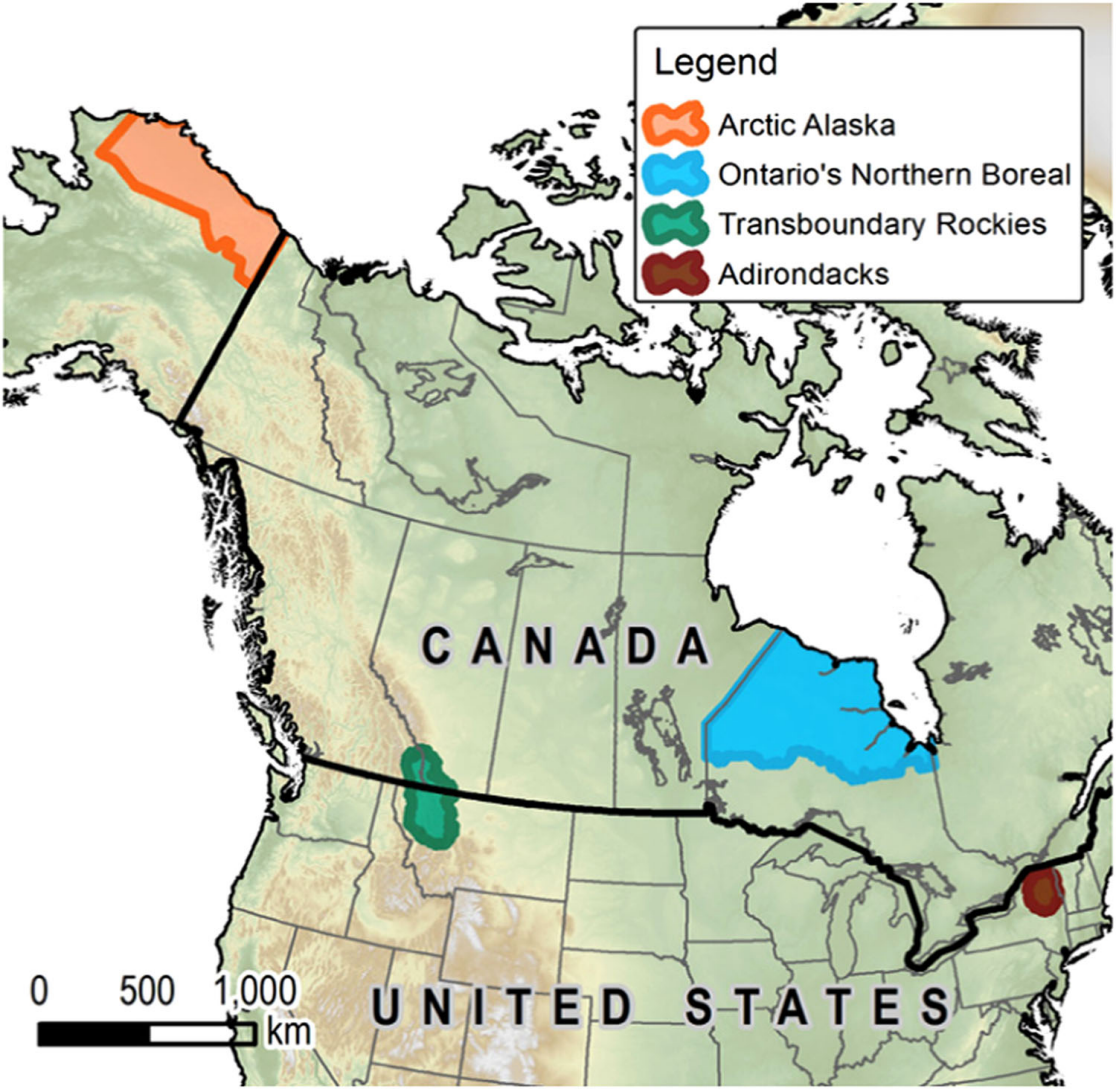
Four North American conservation landscapes selected for this study

To address these research gaps and provide a more nuanced look at barriers and opportunities affecting planning and implementation of adaptation actions to conserve biodiversity, we conducted a comparative study across four North American landscapes: northern Ontario (Canada), the Adirondack State Park (US), Arctic Alaska (US), and the Transboundary Rocky Mountains (US and Canada). These landscapes include two countries (Canada, USA), three states (New York, Alaska, Montana) and one province (Ontario), and each face different challenges due to varied climate change trajectories, political contexts, and social and ecological systems (Fig. 1, Table 1). By ranking barriers and opportunities according to perceived importance, and quantifying similarities and differences across the four landscapes, we sought to identify general as well as contextually-dependent factors affecting adaptation in these regions. In researching opportunities as well as barriers, we aimed to go beyond an investigation of what is not working to offer insight on factors enabling practitioners to overcome obstacles and implement adaptation action in each region. Building on a history of significant involvement in conservation in each of the four landscapes, study results and analyses inform work that the Wildlife Conservation Society (WCS), a global conservation non-governmental organization (NGO), engages in with partner organizations to develop practical strategies for advancing climate change adaptation efforts. This study also supports the development of cross-cutting “lessons learned” about climate change barriers and opportunities across regions. The impacts of climate change create increasingly challenging social, political, and ecological conditions across the globe which make determining how to move from planning to action ever more important.

**Table 1:** Comparison of key factors across four priority conservation landscapes

	Arctic Alaska	Yellowstone Rockies	Adirondack Park	Ontario’s Northern Boreal
Rate and immediacy of climate change	Average annual temperature has increased ~0.5 °C/decade since 1949.	Average annual temperature has increased ~0.3 °C/decade since 1951.	Average annual temperature has increased ~0.2 °C/decade since 1951.	Average annual temperature has increased as much as 1.3 °C since 1949.
	Future temperature increases projected to be the most rapid and of the greatest magnitude.	Future temperature increases projected to be higher than global averages.	Future temperature increases projected to be higher than global averages.	Warming and precipitation are expected to increase in the future, particularly in winter.
Likely availability of future refugia for current species and ecosystems	Arctic systems cannot move further poleward, or up in elevation, if conditions in current locations become unsuitable.	Yellowstone Rockies are a mix of species and ecosystems at their southern and northern edges, and the center of their distributions.	The Adirondack Park lies at the transition between southern cool-temperate forests, and northern boreal forests so shifts between these ecosystem types are likely.	If conditions in current locations become unsuitable, sub-arctic systems may be able to move further poleward, across the James and Hudson Bay, but not up in elevation due to a lack of topographic diversity.
	Future refugia within the Arctic contingent on identifying relatively fine-scale climate refugia (e.g., fog-bound coastal areas).	Topographic diversity may allow for higher elevation refugia; species and ecosystems may find refuge at higher latitudes.	Refugia may exist for boreal species further north, but it is unclear whether all species will be able to move there.	
Relative degree of internal ecological integrity and isolation	Highly intact with little development to date.	Relatively intact both internally, and with other large core protected areas.	Relatively intact within the Adirondack Park, but relatively isolated from other large core protected areas.	Highly intact with little development to date.
	Biggest current threat to ecological integrity is mining and energy development.	Biggest current threats to ecological integrity are changing land use and human population growth.	Biggest current threat to ecological integrity is private land development.	Biggest current threat to ecological integrity is mining, energy development and uncertainty in government-to-government negotiations.
Political context	Mix of primarily federal lands, native lands (scattered low density villages), and state lands.	Mosaic of largely federal and private lands, under federal, state, and county jurisdictions.	Mostly publically-owned state and private land, with land use regulated by the Adirondack Park Agency, in coordination with other agencies.	Treaty 9 (1905/06, 1929/30), Crown land (Provincial and Federal, includes 34 First Nation reservations).

## Methods

To solicit perceptions of barriers and opportunities to climate adaptation in these landscapes, we conducted an online survey of decision-makers, scientists, and managers engaged in natural resource management in North America who were invited to participate in a series of climate change workshops hosted by WCS. Surveys were followed by a series of semi-structured interviews with a subset of survey respondents.

### Study Sites

The study focused on four landscapes where WCS maintains a long history of investment in conservation action and on-the-ground scientific research in the US and Canada: northern Ontario (Canada), the Adirondack State Park (US), Arctic Alaska (US), and the Transboundary Rocky Mountains (US and Canada). (Fig. 1).

### Workshops

WCS hosted climate change workshops in each landscape between 2010 and 2012. Workshop participants included employees of federal, state, and provincial agencies or ministries, NGO employees, Indigenous Peoples (specifically, First Nations from Treaty No. 9 in northern Ontario, Canada), and academics. WCS invited individuals to attend these climate change workshops based on their expertize, experience and influence in their respective regions. Some workshop participants were directly engaged in natural resource management and decision-making, whereas others conducted research, education and policy work supporting natural resource management.

### Survey

We sent a web-based questionnaire to all individuals invited to participate in the workshops (n = 165), within two years after the workshops. We chose a web-based survey as it allowed for branching and skipping questions and made it possible to tailor a single survey to four regions (Sexton et al. 2011). We contacted respondents at their work e-mail addresses and used a standard four-wave design (Dillman 2000). The questionnaire was divided into two main parts: (1) a workshop evaluation for those participants who attended, including a segment on adaptation action taken since each meeting; and (2) a section for all respondents to rank the perceived level of importance of various barriers and opportunities affecting adaptation in practice, with a specific section on collaboration. In this paper, we present the results from the second part of the survey.

We developed the questionnaire (Appendix 1) based on a review of evaluations after each workshop, a review of the climate change literature, and discussions with natural resource professionals in order to create a list of potential barriers and opportunities to adaptation practice. To elicit responses uninfluenced by the content of forced-choice questions, sections on barriers and opportunities began with open-ended questions on climate change. We provided lists of barriers and opportunities and asked participants to rate the current importance of each in affecting progress made by them, or their organizations, in addressing climate change.

### Interviews

We conducted ten semi-structured interviews after the survey was administered to further investigate collaboration, progress within the phases of climate change adaptation, and highly ranked barriers and opportunities (Appendix 2). From a pool of survey respondents who indicated a willingness to participate in interviews, we selected participants to achieve a balance among regions, employment groups, and gender. Interviews were conducted by phone, ranged in length from 60 to 90 min, and were voice recorded with consent.

### Data Analyses

We analyzed survey data using SPSS 20 (IBM Corp. 2011). For barriers and opportunities, rated by participants on a four-point importance scale (“not at all important”, “somewhat important”, “important”, and “very important”), we calculated scores using only “very important” responses. We ranked barriers and opportunities for combined regions as well as for individual regions. Where “very important” scores were equal between barriers or opportunities, we used “important” scores to break the tie. For results and discussion, we refer to rankings instead of raw scores to show how barrier scores compared to one another within each landscape. We coded open-ended questions to identify patterns and themes, and ranked the themes according to their frequency in responses. We transcribed and coded interviews according to dominant themes identified in the survey. We present combined results for the four landscapes, unless stated otherwise, and results based on “rankings” refer to quantitative survey findings.

Our analyses are based on a census of individuals invited to WCS workshops. Of the survey respondents, 64% were workshop participants; 36% were invited to the workshops, but did not participate. The individuals invited to participate in the workshops were identified by WCS as already having an interest in climate change adaptation and/or as influential actors in each landscape. Due to the non-random sampling used to select the study regions as well as the survey and interview participants, results from this study may not be generalizable across all agencies, organizations or regions; yet, experiences and lessons learned can inform climate change priorities in these landscapes and should have application for other organizations addressing climate change in similar types of landscapes.

## Results

### Baseline Information

**Fig. 2:**
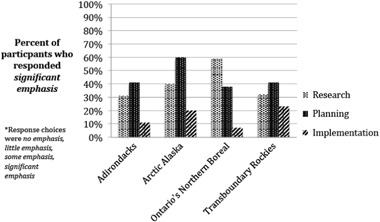
Regional representation of emphasis placed on climate change research, planning and implementation

Of the 165 workshop invitees asked to complete the survey, 97 (59%) completed the entire survey, a typical result for a setting in which participants are vested (Dillman 2000). Thirty-five percent of completed surveys were from the Adirondacks, 29% from Ontario’s Northern Boreal, 22% from the Transboundary Rockies, and 14% from Arctic Alaska. Across all landscapes, 87% of participants responded that climate change is part of their organization’s mission. Combined regional responses showed that 43% of organizations place significant emphasis on planning, 40% place significant emphasis on research, and 14% place significant emphasis on implementation. Emphasis on these elements varied across regions (Fig. 2).

### Barriers to Climate Change Adaptation

**Fig. 3:**
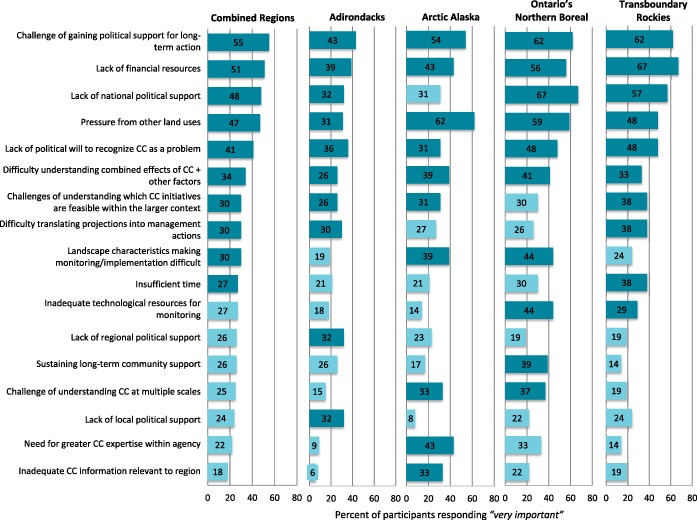
Regional ranking of barriers. Top ten barriers in each region shaded in dark blue

Several broad themes on barriers emerged including: lack of political support for climate change work, resource deficits, the challenge of identifying management options to address the impacts of climate change, and pressures from and complexities related to land uses and their interactions with climate change (Fig. 3). We include qualitative statements from study participants. Open-ended survey responses on barriers largely coincided with those found in forced-choice survey results, and interview responses about barriers supported survey findings and provided a nuanced understanding of top barriers from the survey results.

#### Lack of political support and leadership

Three of the most highly ranked barriers in the survey related to lack of political support (Fig. 3). “The challenge of gaining political support for long-term action” was ranked either first or second within each landscape, making it the only barrier to consistently rank that high across all four landscapes. Several respondents also highlighted the influence of shifting political priorities on their ability to carry out long-term climate change work:

*The priorities shift with changing Administrations and that makes the maintenance of a good solid long term monitoring program, for instance, challenging.*

*~Federal agency employee, Arctic Alaska*

Insufficient political support for climate change was the second most common barrier in the open-ended responses, mentioned by 32% of the respondents (*n* = 85). Interview participants echoed the relative importance of a lack of political leadership and political will:

*And not sure it’s fair to say that it’s funding or lack of data that is a… hindrance… certainly those things are in some cases, but really this has been due to the lack of political will.*

*~State agency employee, Adirondacks*

With the exception of “gaining political support for long-term action,” Arctic Alaska ranked political barriers lower than did the other regions (Fig. 3). Political barriers were more prevalent in the Adirondacks than any other region (Fig. 3).

#### Resource deficits

Quantitative results identified several highly ranked barriers related to lack of resources, including deficits in funding, expertise, and appropriate technology (Fig. 3). Lack of financial resources was also mentioned most frequently (48%, *n* = 85) in the open-ended survey section and highlighted by interview participants:

*There are…approaches to make forests more robust to climate change, but all of them have relatively high costs—none of them actually makes money for the agency. We have no extra capital to spend on new projects, regardless of their merits.*

*~Federal agency employee, Transboundary Rockies*

Lack of staff and related time constraints were the fourth most mentioned barrier in open-ended responses (16%, *n* = 85) and figured prominently in interviews, particularly with state, provincial and federal agency employees, who identified staff cuts and lack of human capacity as significant barriers:

*Our staff have a million things to do and, and [I have] 100 less staff than I had 5 years ago to do it.*

*~State agency employee, Adirondacks*

While funding deficits were considered important in all regions, lack of financial resources was ranked higher in the Transboundary Rockies than in any other region. Ontario’s Northern Boreal respondents considered lack of financial resources as relatively less important than other barriers (Fig. 3). Other types of resource deficits emerged as important in individual regions, such as “Need for greater expertise,” which ranked fourth in Arctic Alaska and “Inadequate technological resources,” which ranked sixth in Ontario’s Northern Boreal (Fig. 3).

#### The challenge of identifying management actions to address the impacts of climate change

Defining appropriate adaptation management actions was identified as a barrier across all regions, with “Determining feasible climate change initiatives within the broader context” and “Difficulty of translating projections into actions” ranking seventh and eighth, respectively (Fig. 3). Open-ended responses reinforced this challenge:

*There is still… a big disconnect between, “okay, now we notice,” [and] “what do we do about it?”*

*~Federal agency employee, Transboundary Rockies*

The difficulty of translating projections ranked in the top ten in the Adirondacks and the Transboundary Rockies, and determining feasible initiatives appeared in the top ten barriers of all regions, except Ontario’s Northern Boreal (Fig. 3).

#### Competing land uses

Two highly ranked barriers in combined regions arise from the multiple uses and anthropogenic factors affecting rural and wild landscapes: “Pressure from other land uses” (ranked fourth overall and first in Alaska) and “Understanding the combined effects of climate change and other factors” (ranked sixth) (Fig. 3). Interview participants also emphasized these factors:

*There’s a very much acknowledged focus on climate change that’s affecting Alaskan resources. Where it breaks down…is…in the consideration of the additional or cumulative factors that [with] climate change may exacerbate sensitivity of wild lands.*

*~Federal agency employee, Arctic Alaska*

### Opportunities for Climate Change Adaptation

**Fig. 4:**
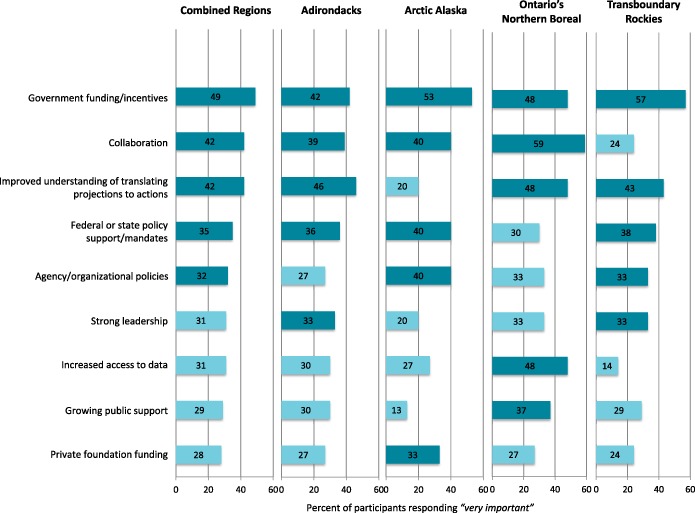
Regional ranking of opportunities. Top five opportunities in each region shaded in dark blue

Overall, themes that emerged from surveys and interviews about opportunities related to adaptation included resource availability, political support and public concern, collaboration, improved ability to select management actions to address climate change, recognition of climate change as a key conservation issue, and leadership (Fig. 4). Compared to our results on barriers, we found quantitative and qualitative results for opportunities differed more.

#### Resource availability

Although lack of financial resources was considered one of the most influential barriers (Fig. 3), “Government-sponsored funding or incentives” was a top-ranked opportunity (Fig. 4). The perceived availability of funding for climate change adaptation was the second most common open-ended response on opportunities (17%, *n* = 86):

*Overall, funding is tight and the economic situation being what it is in the United States…everybody’s feeling the pinch…But that said, if you have a distinct climate change focus, I think it increases your chance of getting funding…we’re always…incorporating that climate change aspect into our proposals …to secure adequate funding.*

*~Federal agency employee, Arctic Alaska*

Availability of funding was ranked first or second in all regions, except Ontario’s Northern Boreal, where it ranked fourth. Private foundation funding ranked within the top five opportunities for Arctic Alaska only.

#### Political support and public concern

Political context, like financial resources, emerged as a barrier and an opportunity in our study. Funding from government sources was quantitatively the highest ranked opportunity (Fig. 4), while “Policy support and/or mandates” ranked fourth (Fig. 4). Participants mentioned political support as an opportunity and noted specific political mandates driving climate change work in their agency/organization:

*The most significant factors that are actually driving institutional change are pressure from Washington.*

*~Federal agency employee, Transboundary Rockies*

Public concern about climate change was the third most common theme in open-ended responses regarding top opportunities across regions:

*Increased recognition by stakeholders e.g. the public, First Nation communities that climate change is a real threat to current lifestyles.*

*~Academic, Ontario’s Northern Boreal*

Political support as a perceived opportunity of climate change adaptation ranked lowest in Ontario’s Northern Boreal and highest in Arctic Alaska (Fig. 4). “Growing public support” ranked fifth in Ontario’s Northern Boreal. While it was not ranked in the top five opportunities of other landscapes, it appeared frequently across landscapes in open-ended responses.

#### Collaboration

“Collaboration between organizations/agencies” was the second highest ranked opportunity (Fig. 4). All interview participants stressed collaboration as essential to addressing the impacts of climate change. Some emphasized that collaboration is expected in all types of conservation work, including climate change adaptation, and others highlighted how collaboration brings together expertise and resources:

*…as a small group of scientists, it is also important to try and maximize our impact by bringing people together with expertise, decision-making authorities, and different kinds of knowledge…*

*~NGO employee, Ontario’s Northern Boreal*

Collaboration received the highest score in Ontario’s Northern Boreal, in contrast to the Transboundary Rockies, where it received the lowest score (Fig. 4).

#### Improved ability to select management actions to address climate change

“Improved ability to translate climate change projections into management action” was the third ranked opportunity across landscapes (Fig. 4). Several interview participants reported it as an area of growth for their organization/agency:

*[Our ability to use climate change data to make decisions] has gotten 300 times better. I think we’re getting better and better at…looking at the different machinations of how to analyze [climate change data] and how you can use it.*

*~Federal agency employee, Transboundary Rockies*

“Translate projections into management actions” ranked first or second in all landscapes, except Arctic Alaska, where it did not appear in the top five opportunities (Fig. 4).

#### Climate change champions and recognition of climate change as an important issue

“Policies within your agency/organization” and “Strong leadership” were ranked the fifth and sixth most important opportunities (respectively) across all landscapes (Fig. 4). Individual and/or organizational recognition of climate change as a critical issue was mentioned twice more often than other opportunities in open-ended results (43%; *n* = 85):

*Within our territory, climate change affects the waters, wildlife, birds, landscape.*

*~First Nations citizen, Ontario’s Northern Boreal*

Participants also frequently mentioned leadership at an organizational level as a driving influence on adaptation efforts:

*There is an executive order that is out there that says “We’ll build it into everything that we do,” but nobody asks about it. They’re not staffing us…they’re not saying “get out there and do more” so I’m the one who’s pushing us to build it into the very things that we do…Particularly when you’re doing significant paradigm shifts and rethinking how we do things…Getting people to think about where we’re going instead of where we’ve been…[leadership] is very important*.

*~State agency employee, Adirondacks*

Strong leadership ranked in the top five in the Transboundary Rockies and the Adirondacks (Fig. 4), and was mentioned frequently in survey and interview responses across landscapes. Organizational policies encouraging climate change work ranked in the top five opportunities in the Arctic Alaska and Transboundary Rockies landscapes only (Fig. 4), but individual and organizational recognition of climate change as a key issue figured prominently in open-ended responses from all landscapes.

## Discussion

Although 87% of participants in this study indicated that addressing the impacts of climate change is part of their organization’s mission or mandate, only 14% reported significant emphasis on the implementation of climate change adaptation. Our results provide insight into the most important barriers and opportunities to adaptation as perceived by a variety of managers, researchers, private landowners, Indigenous Peoples, and decision-makers in four North American regions and enable us to identify regional differences and similarities to further refine recommendations and influence adaptation action in these and other landscapes.

### Regional Similarities

Across the four landscapes, managers face common barriers to advancing adaptation, including lack of political support, resource deficits, competing pressures from land use changes, and uncertainty in designing management actions to address the impacts of climate change. Resource deficits, particularly funding, are frequently cited as a primary explanation for why practitioners have not begun implementation of adaptation actions (Moser and Ekstrom 2010; Archie et al. 2012; Kemp et al. 2015). While 79% of survey participants in this study considered lack of financial resources an important barrier, other barriers emerged as more influential in most landscapes, an indication that participants perceive more diverse challenges to adaptation. In particular, participants in all the landscapes but Arctic Alaska identified lack of political will and support as a top impediment to adaptation, particularly as it related to initial problem recognition, priority setting and allocation of resources. This finding is consistent with the international literature for urban and rural settings, which shows that a lack of high level government support can inhibit climate change adaptation efforts at local scales (Measham et al. 2011; Aylett 2015; Lemieux et al. 2015). In our study, survey respondents in every landscape identified the challenge of sustaining political support for long-term action as one of the top two most influential barriers to adaptation action. Similarly, participants highlighted the difficulty of addressing the scale and complexity of climate change within the constraints of one or two-year funding cycles. Taken together, our results support other findings that suggest the short-term nature of political agendas and funding cycles has significant effects on an organization’s capacity to implement actions to address climate change (Crabbé and Robin 2006; Ford et al. 2011; Bierbaum et al. 2013; Aylett 2015).

Barriers related to climate change data and information, such as uncertainty of climate change projections and poor information transfer between researchers and practitioners are well-documented (Jantarasami et al. 2010; Murthy et al. 2010; Kemp et al. 2015); however, these particular barriers did not emerge as influential in our study suggesting there has been progress in making climate change information more readily available, in these landscapes at least. More significant and pervasive across all landscapes were barriers related to understanding how climate change interacts with complex social, ecological and political dynamics, ongoing land uses and other changes affecting wild ecosystems, and identifying appropriate management actions in response to those interactions. This finding reflects the results from Aylett (2015), who found that barriers become more pronounced as one moves from general climate change information to understanding local impacts and formulating definitive management plans. Our interview responses strongly suggest that even when managers accept climate change as a critical threat to resources and ecosystems, the general challenge of ‘not knowing what to do about it’ may prevent them from taking meaningful action (Jantarasami et al. 2010; Archie et al. 2012; Kemp et al. 2015), especially if they simultaneously face other limitations, e.g., funding deficits, staffing and time constraints, and a lack of political support. Such findings indicate that barriers cannot be understood as isolated factors, but as interconnected and cumulative influences on adaptation action (Eisenack et al. 2014).

In addition to perceiving common barriers to climate change adaptation, managers also identified a number of common opportunities that have helped advance climate change planning and action in their regions, including those related to government funding and incentives, collaboration, and leadership. Collaboration emerged in our survey results as being necessary to address the impacts of climate change, a finding supported in the social learning and adaptation literature (Collins and Ison 2009; Lauber et al. 2011; Juhola and Westerhoff 2011; Eisenack et al. 2014). Without exception, interview participants corroborated the critical role that collaboration plays, particularly in enabling work across large landscapes, as well as in overcoming barriers related to resource deficiency by promoting shared resources, information and expertize (Burch 2010). The importance of collaboration is consistent across many natural resource management contexts where governments often formally or informally rely on other entities to achieve desirable management goals (Wondolleck and Yaffee 2000; da Fonseca 2003; Hatchwell 2014). Reflecting these results, a number of organizations have adopted a collaborative and participatory approach to climate adaptation, bringing science and management experts together to learn about climate change science and use systematic planning processes to apply that science to management decisions (Cross et al. 2012; Littell et al. 2012; Cross et al. 2013; Janowiak et al. 2014).

Political and organizational leadership also emerged as critical enablers of adaptation action, a finding consistent with other studies (Ford et al. 2011; Bierbaum et al. 2013; Eisenack et al. 2014). Our study participants identified leadership as important in terms of setting agendas, directing resources, leading collaborative efforts, initiating projects, and sustaining momentum. These findings suggest that leadership can help overcome barriers related to competing priorities, financial and human resource deficits, and lack of long-term support for adaptation. Interview results also suggested that organizational leadership can help close the gap between national policy and adaptation action at the local level, and may be a critical factor in shifting to new approaches and new ways of looking at issues that include climate change (Moser and Ekstrom 2010; Measham et al. 2011; Shi et al. 2015). The above results related to collaboration and leadership support the idea that strengthening the ability to convert existing resources into action can be as critical as finding additional resources (funding, time or information) (Burch 2010).

The finding that particular barriers and opportunities are common across most or all landscapes highlights that certain factors can affect adaptation efforts regardless of the specific context or location. We suggest this finding offers support for the importance of cross-site learning. With comparative studies, the findings may yield more generalizable recommendations or principles for adaptation. For example, investing in efforts that lessen financial constraints, build and strengthen collaboration, and develop and support climate change advocates within organizations and agencies, in local communities, and at various political levels will likely help to promote adaptation action in all landscapes. Yet addressing only these barriers may not be sufficient to move from adaptation planning to implementation, due to the landscape-specific nature of other barriers, as described below.

### Regional Differences

Differences in how participants in our study reported barriers and opportunities across regions lend support for the need to also plan and develop adaptation efforts tailored according to specific contexts and the needs of particular landscapes. For example, while participants in all of the landscapes identified the importance of barriers related to political will and support, those barriers varied across landscapes in their scale and nature. In the Adirondacks, a landscape composed entirely of state and private lands, highly ranked barriers illustrated the need for increased political will and support at local and regional scales. In that landscape, it will likely be necessary to invest in activities that can increase political support for adaptation among the more than 100 towns and villages in the region, as well as within the regional Adirondack Park Agency. However, in landscapes such as the Transboundary Rockies, dominated by federal public lands and managed by federal agencies, investments in political will at the national level may be relatively more important. In Ontario’s Northern Boreal, where First Nations and government ministries are the decision-makers, participants perceived a lack of national political support as an influential barrier, a finding supported in other literature addressing integration of climate change adaptation in higher level environmental planning in Canada (Aylett 2015). Consequently, strengthening formal provincial, federal and international policies and commitments will be important to integrating local approaches to adaptation and supporting internal networks for addressing climate change within Ontario. Understanding how adaptation can best be coordinated across jurisdictions and among different levels of government, and connecting high-level directives to local strategies and efforts will be essential to building, managing, and sustaining effective climate change adaptation in each region.

Disparities among regions in the relative importance of challenges associated with understanding climate change impacts and translating the science into action should lead to differential investments in funding, research and monitoring across the landscapes. For example, in Ontario’s Northern Boreal, investment in technology to better monitor climate and ecosystem changes appears important, while adaptation in Alaska may benefit more from providing Arctic-specific climate change information and strengthening climate change expertize within organizations. In the Transboundary Rockies and Adirondacks, priorities include less emphasis on research related to climate change impacts, and more effort on building practitioners’ comfort level with translating the science that does exist into practical management strategies. The decision-making process for investing in climate change work might benefit from considering more targeted approaches to climate impacts assessment that “first” identify the most critical information needs and the sensitivities of particular systems “before” analyzing climate change projections to evaluate risk (Brown and Wilby 2012). These additional steps could help identify and prioritize appropriate local sites for management actions and evaluate the tradeoffs between multiple management options.

Differences in opportunities among landscapes can also suggest appropriate directions for funding and adaptation efforts. Highly ranked opportunities in a particular landscape lend insight into what should likely receive continued focus. For example, in Ontario’s Northern Boreal, participants rated increased access to data as an influential opportunity whereas participants from Arctic Alaska saw private funding as being important to their adaptation efforts. Opportunities considered less influential by participants may indicate areas that could be targeted to strengthen adaptation efforts in the future.

### Barriers as Opportunities

Several themes that were top barriers across landscapes were also top opportunities, including funding, political support and leadership, and using climate change data to identify management actions. This finding is congruent with other studies showing that certain factors may serve as either drivers or barriers (Engle 2012; Uittenbroek et al. 2012; Lemieux et al. 2015). The presence of particular factors as both barriers and opportunities may indicate that individuals or organizations in our study have been able to transform barriers into opportunities through creativity, learning, leadership, and/or use of varied approaches. In the Transboundary Rockies and Adirondacks, for example, organizations have been able to circumvent political and community wariness of climate change by framing climate change adaptation in terms of key regional resource issues. In the Rockies, framing climate change as a water management issue has garnered support from natural resource management agencies and the ranching community who have implemented actions to address declining snowmelt inputs to streams, such as the installation of low-cost and low-tech structures constructed from willow branches and other local vegetation. These structures mimic beaver activity and restore the natural water storage capacity of riparian and wetland ecosystems. In the Adirondacks, framing of the issue in terms of protecting winter recreation opportunities, a major economic driver in the area, has motivated groups to work together on addressing climate-related issues. For instance, three New York state-owned ski resorts (two based in the Adirondacks) have pledged to be powered by 100 percent renewable energy by 2030 and invest millions of dollars into making the resorts year-round destinations with ample non-snow related activities. Other landscapes and organizations may be able to incorporate similar approaches in their own efforts, corroborating the importance of sharing insights and expertize across regions and between organizations.

## Conclusion

Priorities for conservation action in the face of climate change within a particular region must include an understanding of how climate change interacts with the myriad other forces affecting conservation of wild landscapes, and how adaptation can most effectively be carried out within the social, political, and cultural contexts that influence governance and adaptation decisions in that region. Fortunately, some frameworks have successfully helped practitioners integrate climate change planning into management actions (e.g., Gleeson et al. 2011; Poiani et al. 2011; Cross et al. 2013; Raymond et al. 2013; Janowiak et al. 2014). Some decision-support tools, such as scenario planning (e.g., Weeks et al. 2011; Rowland et al. 2014), are useful for integrating climate change alongside other stressors and land uses, facilitating collaborative planning across disciplines and organizations. Further use of these methods and frameworks may help to address a number of the barriers and opportunities identified by participants in this study. In fact, our results suggest that within some landscapes increased investment in adaptation planning and the translation of existing science into management actions may be more effective at addressing key barriers than would investing in more or ‘better’ science on climate impacts.

By connecting networks of individuals, collaboration should enable identification, education and support of climate change leaders and advocates. In working with targeted advocates, particularly in areas with relatively less political, public or agency/organizational support for climate change, it may be useful to discuss approaches for effectively bringing climate change considerations into discussions and planning sessions with co-workers and organizational leadership. Broadening the reach of climate change planning and educational efforts to include other planning professionals, such as city, municipal/county, and regional planners and local-elected and regionally-elected officials, could also contribute to increasing political support for adaptation. As collaboration is an important tool for understanding local and cultural contexts, as well as for building capacity to mobilize resources and expertize to address complex natural resource challenges (Wondolleck and Yaffee 2000; Bodin and Crona 2009), the effect of collaboration on adaptation planning and implementation will be important to assess throughout the process.

Additionally, sharing experiences across regions using common terms for barriers and opportunities, as presented in this paper and incorporated from other research, will be an important next step for improving adaptation planning and implementation. Several opportunities exist for sharing these lessons, for example through climate adaptation networks and hubs such as the Ontario Centre for Climate Impacts and Adaptation Resources (http://www.climateontario.ca/) and the Northern Institute of Applied Climate Science (https://www.forestadaptation.org). Professional conservation, management and adaptation conferences, such as the National Adaptation Forum (https://www.nationaladaptationforum.org) or the North American Wildlife and Natural Resources Conference (https://wildlifemanagement.institute/conference) offer additional opportunities for exchanging ideas on ways to overcome barriers and capitalize on opportunities to advance adaptation progress.

Overall, our results suggest that there is value in exploring the specific factors that might enable or inhibit adaptation progress within and across diverse landscapes, so that limited time and financial resources are strategically invested to address potential barriers and capitalize on available opportunities.

## Electronic supplementary material

Online Resource 1

Online Resource 2
