# Supplementary material for: Similarities and Differences in Barriers and Opportunities Affecting Climate Change Adaptation Action in Four North American Landscapes
Source: Environ Manage. 2017 Sep 7;60(6):1076–89. doi: 10.1007/s00267-017-0933-1 (PMC5658473; doi:10.1007/s00267-017-0933-1)
Supplement: Supplementary file 1 — Online Resource 1 [file 267_2017_933_MOESM1_ESM.pdf]

Similarities and Differences in Barriers and Opportunities Affecting Climate Change Adaptation Action in Four North American Landscapes

Environmental Management

Authors:

Whitney R. Lonsdale, Cornell University, whitneylonsdale@gmail.com, 828.606.8409, 502 N 9th Ave, Bozeman, MT, 59715

Heidi E. Kretser, Cheryl-Lesley B. Chetkiewicz, Molly S. Cross

Caption: Survey Instrument used for study

Online Resource 1

Climate Change Research, Planning and Action

You have been asked to participate in the following survey because you either participated or were invited to participate in one of the climate change planning workshops organized by Wildlife Conservation Society (WCS) and partners between 2009 and 2011. We greatly appreciate your cooperation. Your input and insight will contribute significantly to our larger goal of working with communities and resource managers to increase ecological and social resilience in a changing climate.

The goals for this survey are to help the Wildlife Conservation Society evaluate the effectiveness of its current workshop model on planning for climate change, and to gain insight into the opportunities and barriers that affect the process of addressing climate change. If you did not attend a workshop, you will be able to skip the workshop evaluation section of the survey.

This survey is only concerned with actions that individuals and organizations are taking to understand or address the **effects of climate change** (e.g., negative or positive impacts) on species, ecological processes or ecosystems of conservation or management interest. It is NOT asking about efforts related to addressing the sources of climate change (e.g., greenhouse gas emissions, energy efficiency).

This survey is a collaborative project between the Wildlife Conservation Society and a Cornell University master's degree student. This survey is funded through a grant from the Kresge Foundation focusing on natural resource management organizations and agencies and the process of addressing climate change.

Your participation is voluntary and confidential. We will never associate your name with your response.

If you have any questions or concerns pertaining to this survey, please contact:

XXXXX
Cornell University
XXXXX

XXXXX
Wildlife Conservation Society
XXXXX

XXXXX
Cornell University
XXXXX

THANK YOU FOR YOUR HELP!

PART 1: REGIONAL AND ORGANIZATIONAL FOCUS ON CLIMATE CHANGE

Please indicate your response by clicking on the box corresponding to your answer, or fill in the blank box with the appropriate information.

Reminder: This survey is only concerned with actions that individuals and organizations are taking to understand or address the **effects of climate change** (e.g., negative or positive impacts) on species, ecological processes or ecosystems of conservation or management interest. It is NOT asking about efforts related to addressing the sources of climate change (e.g., greenhouse gas emissions, energy efficiency).

1. Is addressing the effects of climate change part of your organization's mission?
☐ Yes; ☐ No
IF YES, GO TO #2; IF NO, GO TO #3

2. How long has climate change been part of your organization's mission?
☐ *0-1 yrs;* ☐ *1-2 yrs;* ☐ *3-4 yrs;* ☐ *5-7 yrs;* ☐ *8-10 yrs;* ☐ *more than 10 yrs*

3. In your opinion, please specify the amount of emphasis, in terms of formal goals and resource allocation, placed by your organization on each of the following elements of addressing the effects of climate change.

	Please check one box for each choice			
	Significant Emphasis	Some Emphasis	Little Emphasis	No Emphasis
Research/Data Collection/ Monitoring – *Collect information on climate changes, climate change effects on ecosystems, or other climate change-related information*	☐	☐	☐	☐
Planning *–Identify and prioritize strategies for addressing the impacts of climate change*	☐	☐	☐	☐
Implementation – *Execute planned strategies to address climate change*	☐	☐	☐	☐
Other ☐	☐	☐	☐	☐

4. In your opinion, has your organization prioritized climate change in its research, planning, and action due to pressure from any of the following bodies:

Please check all that apply

Federal government	☐ *Yes* ☐ *No* ☐ *Don't know*
State government	☐ *Yes* ☐ *No* ☐ *Don't know*
The organization itself	☐ *Yes* ☐ *No* ☐ *Don't know*
Private funding sources and public grantors	☐ *Yes* ☐ *No* ☐ *Don't know*
Local community	☐ *Yes* ☐ *No* ☐ *Don't know*

5. In your opinion, what percentage of the staff in your organization considers climate change a top priority issue?

☐ *0-25%*
☐ *26-50%*
☐ *50-75%*
☐ *76%-100%*

6. What percentage of the people in the communities or areas you serve believes that climate change is a top priority issue?

☐ *0-25%*
☐ *26-50%*
☐ *50-75%*
☐ *76%-100%*

7. Did you participate in a WCS climate change planning workshop in 2009 or 2010?

☐ *Yes*
☐ *No*

IF YES, SKIP TO #9; IF NO GO TO #8

8. Please state your reason for not attending the workshop.

☐ *Travel restrictions*
☐ *Scheduling conflict*
☐ *Time constraints*
☐ *I was personally not sufficiently interested*
☐ *My supervisor was not sufficiently interested*
☐ *Lack of funding*
☐ *Other*

(FROM HERE SKIP TO PART 3 OF THE SURVEY)

9. In which climate change planning workshop did you participate?

☐ Northern Rockies, 2010, Fernie, focused grizzly bears and wolverines in the US/Canada Transboundary Region

☐ Adirondacks Workshop, November, 2010 at Blue Mountain Lake, focused on lowland boreal wetlands

☐ Arctic Workshop 2009, April 2009, focused on new conservation priorities for a changing Arctic Alaska

QUESTIONS #18 AND #19 WILL BE DIFFERENT DEPENDING ON WHICH WORKSHOP THEY ATTENDED. THERE ARE THREE SETS OF INITIATIVES TO BE USED – ONE FOR EACH WORKSHOP.

PART 2: CLIMATE CHANGE WORKSHOP EVALUATON

The following section focuses on evaluation of the WCS climate change workshop you attended and progress made since the workshop on research, planning and action related to addressing the impacts of climate change

**Workshop Evaluation**						
Please indicate your level of agreement with the following statements:	Strongly disagree	Disagree	Neither agree or disagree	Agree	Strongly agree	Not applicable
10. The science and climate change information presented was relevant to the work conducted by my organization.	☐	☐	☐	☐	☐	☐
11. The workshop helped me to understand how to move from analysis of climate change impacts to the creation of specific conservation strategies and actions aimed at addressing those impacts.	☐	☐	☐	☐	☐	☐
12. After the workshop, I had a clearer understanding of which management and/or monitoring initiatives my organization should prioritize in light of climate change.	☐	☐	☐	☐	☐	☐
13. Since the workshop, projected climate						

change scenarios have factored more importantly into my work.	☐	☐	☐	☐	☐	☐

	Please check one box				
	Not at all	Once or twice	Occasionally	Frequently	Not applicable
14. Since the workshop, I have discussed incorporating climate change effects into planning in my area.					
**With my supervisor**	☐	☐	☐	☐	☐
**With co-workers within my organization**	☐	☐	☐	☐	☐
**With partners outside my organizations**	☐	☐	☐	☐	☐
**With private stakeholders in my region**	☐	☐	☐	☐	☐

**15. Collaborative Opportunities Provided By Workshop**	Please check one box				
Please indicate your level of agreement with the following statements:	Strongly disagree	Disagree	Neither agree or disagree	Agree	Strongly agree
Through relationships formed or strengthened at the workshop I have been able to identify new opportunities for funding, research, and/or collaborative action	☐	☐	☐	☐	☐
Through relationships formed or strengthened at the workshop I have been able to gain new understanding of climate-change related topics	☐	☐	☐	☐	☐

16. Did the workshop change the way you think about or apply climate change information in your work? ☐ Yes ☐ No

(IF YES GO TO #17, IF NOT, GO TO #18)

17. How did the workshop change the way you think about or apply climate change information in your work?

18. Has your organization followed up on any of these next steps or recommendations identified at the workshop? *Please check "yes" or "no" for each*

INSERT HERE INITIATIVES FROM EACH OF THE THREE WORKSHOPS

EACH INITIATIVE THEY MARK AS A "YES" IN #18 WILL DROP DOWN SO THEY CAN FILL IN THE CHOICES IN #19 FOR EACH

19. For each initiative, please check the choice that most closely matches the manner of progress made.

☐ *Further informal discussion,*
☐ *Further formal discussion and/or planning*
☐ *Action plan developed*
☐ *Actions initiated*
☐ *Measurable outcomes demonstrated*
☐ *Other*

This concludes the section of the survey focused on evaluation of the climate change workshops in which you participated.

PART 3: COLLABORATION IN ADDRESSING THE EFFECTS OF CLIMATE CHANGE

The following two parts of the survey investigate collaboration, as well as barriers and drivers that influence work on climate change-related issues.

Reminder: This survey is only concerned with actions that individuals and organizations are taking to understand or address the **effects of climate change** (e.g., negative or positive impacts) on species, ecological processes or ecosystems of conservation or management interest. It is NOT asking about efforts related to addressing the sources of climate change (e.g., greenhouse gas emissions, energy efficiency).

Questions #20 and #21 do not refer to direct workshop outcomes, but instead investigate the general potential benefits and costs of collaboration in terms of addressing the effects of climate change.

ITEMS FOR WHICH PARTICIPANTS CHOOSE "Has experienced benefit" NEED TO DROP DOWN TO THE FOLLOWING QUESTION WHERE THEY WILL BE ASKED THE IMPORTANCE SCALE.

**20. Potential Benefits of Collaboration**		
Please indicate whether your organization has or has not experienced each benefit listed below.	Has experienced benefit	Has not experienced benefit
Sharing of skills/expertise on climate change	☐	☐
Ability to act effectively at the scale of a large landscapes to address effects of climate change	☐	☐
Access to new funding for addressing effects of climate change	☐	☐
New insights into methods, approaches, and/or priorities for work on climate change	☐	☐

Please indicate the importance of each in terms of increasing the capacity for addressing the effects of climate change.

Not at all important	Somewhat important	Important	Very important
☐	☐	☐	☐
☐	☐	☐	☐
☐	☐	☐	☐
☐	☐	☐	☐

SAME INSTRUCTIONS AS FOR #20

**21. Potential Costs of Collaboration**		
Please indicate whether your organization has or has not experienced each cost listed below.	Has Experienced	Has not experienced
Increased competition for funding to address effects of climate change	☐	☐
Decreased clarity on management goals to address effects of climate	☐	☐

change		
Decreased efficiency in decision-making due to greater number of voices and desire for consensus	☐	☐

Please indicate the importance of each as an impediment to addressing the effects of climate change.

Not at all important	Somewhat important	Important	Very important
☐	☐	☐	☐
☐	☐	☐	☐
☐	☐	☐	☐
☐	☐	☐	☐

22. In your opinion, how important is collaboration between agencies and organizations to addressing climate change?

☐ *Not at all important;* ☐ *Somewhat important;* ☐ *Important;* ☐ *Very important*

PART 4 – OPPORTUNITIES AND BARRIERS

The goal of Part 4 is to investigate both barriers and opportunities that influence work on addressing the effects of climate change on conservation or management targets.

For the sake of this survey, **opportunities are defined as factors that encourage or support progress on addressing the effects of climate change, while **barriers** are defined as factors that slow, alter, or prevent progress on addressing the effects of climate change.

Section 1 – Open-ended Response

23. In your opinion, what are the most significant factors driving agencies and organizations to take action on climate change research, planning and/or action?

24. In your opinion, what are the most significant barriers impeding agencies and organizations from taking action on climate change research, planning and/or action?

Section 2 – Opportunities

Listed below are a number of opportunities that may help organizations and agencies make progress in addressing the effects of climate change.

OPPORTUNITIES	**Current** importance of opportunity			
*Please rate how important each opportunity is **currently** to your organization's efforts to address the effects of climate change.*	Not at all important	Somewhat important	Important	Very Important
Private/ foundation funding for climate change initiatives	☐	☐	☐	☐
Government-sponsored funding or incentives for climate change initiatives	☐	☐	☐	☐
Policy support and/or mandates on a federal or state level	☐	☐	☐	☐
Policies within your agency/organization that encourage work on climate change issues	☐	☐	☐	☐
Collaboration between organizations	☐	☐	☐	☐

Strong leadership within my organization/agency or partner organizations/agencies on taking action to address the effects of climate change	☐	☐	☐	☐
Growing public support for addressing the effects of climate change	☐	☐	☐	☐
Increased access to climate change-related data and information	☐	☐	☐	☐
Improved understanding of how to translate climate change projections into species- or ecosystem-relevant management or conservation actions	☐	☐	☐	☐
Creation of climate change-focused position(s) within my organization	☐	☐	☐	☐
Other ______	☐	☐	☐	☐
Other ______	☐	☐	☐	☐

Section 3 – Barriers to Climate Change Adaptation

Listed below in 8 categories are a number of potential barriers that may alter or impede work on addressing the effects of climate change.

*Please rate how important each barrier is **currently** in slowing or preventing action by your organization to address the effects of climate change*	**Current** importance of barrier			
**CLIMATE CHANGE UNCERTAINTY**	Not at all important	Somewhat important	Important	Very Important
Uncertainty of climate change projections	☐	☐	☐	☐
Inadequate climate change information relevant to specific region	☐	☐	☐	☐
The challenge of understanding climate change at multiple scales or across large landscapes	☐	☐	☐	☐
The lack of information transfer between researchers and practitioners	☐	☐	☐	☐
The difficulty of translating climate change projections into species- or ecosystem-relevant management or conservation actions	☐	☐	☐	☐

RESOURCE AVAILABILITY	Not at all important	Somewhat important	Important	Very Important
The lack of financial resources to address climate change	☐	☐	☐	☐
Insufficient time to address climate change issues	☐	☐	☐	☐
Inadequate technological resources for monitoring vulnerable landscapes and species	☐	☐	☐	☐
The need for greater climate change expertise within organization/agency	☐	☐	☐	☐
**ORGANIZATIONAL SUPPORT**	Not at all important	Somewhat important	Important	Very Important
The lack of support within my own agency/organization for climate change	☐	☐	☐	☐
The need for greater leadership within my organization/agency for climate change planning and implementation	☐	☐	☐	☐
Planners and resource managers being tired of talking about climate change	☐	☐	☐	☐
Planners and resource managers not believing in climate change	☐	☐	☐	☐
**COMPLEXITY & DECISION MAKING**	Not at all important	Somewhat important	Important	Very Important
The challenge of agreeing on priority actions	☐	☐	☐	☐
The difficulty of understanding the combined effects of climate change and other factors (e.g. land use changes driven by economic or social factors) on target landscapes and species..	☐	☐	☐	☐
The challenge of understanding which climate change initiatives are feasible within the political, economic and social context.	☐	☐	☐	☐
Resistance to re-assessing decisions and policies already in place	☐	☐	☐	☐
**POLITICAL SUPPORT**	Not at all important	Somewhat important	Important	Very Important

The lack of political will to recognize climate change as a problem	☐	☐	☐	☐
The lack of political support at the **local** level for addressing climate change	☐	☐	☐	☐
The lack of political support at the **regional** level for addressing climate change	☐	☐	☐	☐
The lack of political support at the **national** level for addressing climate change	☐	☐	☐	☐
The challenge of gaining political support for long-term action	☐	☐	☐	☐
**LOCAL COMMUNITY VALUES**	Not at all important	Somewhat important	Important	Very Important
Local community values and/or beliefs preventing acceptance of climate change as a high priority issue	☐	☐	☐	☐
The challenge of sustaining long-term support for climate change-related initiatives within community	☐	☐	☐	☐
**COLLABORATION**	Not at all important	Somewhat important	Important	Very Important
Lack of collaboration among agencies and organizations for initial research and/or planning	☐	☐	☐	☐
Insufficient collaboration between agencies/organizations and private stakeholders to implement selected actions and/or support long-term action	☐	☐	☐	☐
**LANDSCAPE ELEMENTS**	Not at all important	Somewhat important	Important	Very Important
Landscape characteristics, such as size, accessibility, or inadequate infrastructure, making monitoring and implementation of conservation or management actions difficult	☐	☐	☐	☐
Pressure from other land uses such as energy development or real estate development that make implementation of conservation or management actions difficult	☐	☐	☐	☐

**OTHER**<br>Please use this space to add other barriers not listed above	Not at all important	Somewhat important	Important	Very Important
	☐	☐	☐	☐
	☐	☐	☐	☐

Please check the box that best describes your employment

NGO ☐
State Agency ☐
Federal Agency ☐
Private Consulting ☐
Academic ☐
Other

Please choose the option that best describes your primary job responsibility

Research ☐
Planning/Management ☐
Advocacy ☐
Education ☐

This survey is the first phase of the study. Interviews will be conducted in the spring of 2012 to investigate barriers to planning for climate change in your work. Would you be willing to participate in these interviews?

Yes ☐
No ☐

If yes,

Name:

Phone:

Email:

If you have additional comments to make about planning for climate change or the workshop please type them here

THANK YOU FOR PARTICIPATING IN THIS SURVEY
