# Supplementary material for: Similarities and Differences in Barriers and Opportunities Affecting Climate Change Adaptation Action in Four North American Landscapes
Source: Environ Manage. 2017 Sep 7;60(6):1076–89. doi: 10.1007/s00267-017-0933-1 (PMC5658473; doi:10.1007/s00267-017-0933-1)
Supplement: Supplementary file 2 — Online Resource 2 [file 267_2017_933_MOESM2_ESM.pdf]

Similarities and Differences in Barriers and Opportunities Affecting Climate Change Adaptation Action in Four North American Landscapes

Environmental Management

Authors:

Whitney R. Lonsdale, Cornell University, whitneylonsdale@gmail.com, 828.606.8409, 502 N 9th Ave, Bozeman, MT, 59715

Heidi E. Kretser, Cheryl-Lesley B. Chetkiewicz, Molly S. Cross

Caption: Interview Instrument used for study

Online Resource 2

Hello this is XXXX from Cornell University. We're scheduled for an interview right now, is this still a good time?

Just as a reminder: The purpose of the interview is to further discuss the barriers and opportunities to addressing climate change. I am collaborating with WCS, but I am not a WCS employee and these interviews are part of my Master's thesis research. Your name will never be associated with your responses. Do I still have your permission to record the interview?

There are a number of questions I will ask everyone, but I will also adjust some questions based on the interviewee's job and region. So that I can do this effectively, will you start by telling me briefly about your position?

I. Baseline Questions: I would like to know more about your work as it relates to climate change.

A. Are you currently working on climate change related projects? If Yes, B; If No, C
B. Please briefly tell me about those projects.
C. Are you being asked to incorporate climate change into any part of your day-to-day work?
D. To what extent is climate change a part of your day-to-day work?

II. Barriers – As stated initially, we are particularly interested in what barriers and opportunities exist for implementing climate change related projects. I would like to ask some questions specific to the responses we received on the survey.

A. Funding: Respondents ranked the amount of funding available as a top opportunity and a lack of funding as a top barrier.

1. How does funding influence your work on climate change related projects or issues?
That's my overall topic, let's get to some specific questions

a) Is there a lack of funding overall or a lack of funding specific to climate change related work?
b) Is there a specific aspect of climate change work that is more lacking in funding than others? For example – research, planning or implementation?
c) What factors influence the availability of funding in your organization for climate change work?
d) If additional funding became available to your organization in the next year, what would you suggest it be directed toward? (this could be a climate-change related initiative or something else)

2) In general, is there a process through which your organization allocates funds for climate change related work?

a) Does the kind of data or information you have, or the quality, influence the allocation of funds?
b) How does the political climate affect the allocation of funds?
c) How does leadership influence the allocation of funds?
d) Do you think that overall the allocation of funding matches your organization's priorities?

B. Time - Respondents also ranked lack of time as a top barrier.

1) Within your organization or office, how is it decided how much time is spent on different projects?
2) How much leeway do you as an individual have to choose priorities?
3) What factors affect how you prioritize your time?
4) Are you personally prioritizing climate change as opposed to other things?

C. Translating projections to action: One of the barriers that ranked highly in the survey results was "*The difficulty of translating climate change projections into species- or ecosystem-relevant management or conservation actions.*" This barrier contains two issues: a potential lack of clarity in the climate change data and information, and the challenge of deciding what management actions should be taken in light of available climate change information. The next set of questions will deal with both of these issues.

1) How would you describe your understanding of climate change impacts, present and future, on the system in which you work?
2) How would you describe the quality of information you currently use to make decisions in your work on addressing climate change?
3) How would you describe your experience using the data available to make management choices (or recommendations) to address climate change? (*Alternately, if additional prompting needed:* What challenges

have you experienced in using climate change data to make management choices?)

D. Combined Effects - *The difficulty of understanding the combined effects of climate change and other factors (e.g. land use changes driven by economic or social factors) on target landscapes and species* was also ranked as a top barrier in the survey.

1) Is this barrier a factor in the landscape in which you work?
2) How would you describe its impact on your work in addressing climate change?

E. Comparison of uncertainty and lack of resources – We have already spoken separately about two of the top barriers: the uncertainty of climate change information and data and a lack of resources, specifically time and funding. Now I will ask you to think about these two barriers in comparison to each other.

Which do you think is a more significant barrier - uncertainty or the lack of resources? Comments?

F. Long-term political support – I would like to ask you about one final barrier that ranked highly in the survey results, and that is: *The challenge of gaining political support for long-term action.* The first question is:

1) How do politics influence the priorities of your agency/organization?
2) What level of political arena is most influential - local, state, federal?
3) Has your experience with gaining support for long-term action on climate change been different from gaining support for short-term action?
4) How does this experience differ from gaining long-term support for other conservation issues?

That concludes the section of questions regarding barriers to addressing climate change. I have several more questions for you, which should take about ten minutes.

III. Collaboration - We are interested in investigating collaboration between organizations on climate change-related issues – both the process and the outcomes.

1) Please describe your experience collaborating with other organizations on climate change-related projects.
2) What influences how and when your organization collaborates with other organizations?
3) Is collaboration different for climate change-related issues compared to other conservation issues?

IV. Further Action - We are also interested in investigating what factors influence progress your organization is making on specific climate change-related initiatives.

1) Are there any / What are the primary (specific) climate change-related initiatives on which your organization (or office) has made progress in the past year?
2) What factors were important in enabling that progress?

Prompts if necessary: *high priority for organization, low financial constraints, strong policy support, collaborative efforts, strong leadership, good fit with other conservation initiatives*

My final question for you concerns the process of addressing climate change:

V. Stages - Some research suggests that addressing climate change happens in three distinct stages: understanding the issue, planning, and management, which includes implementation and monitoring.

1) To what extent does your work happen in stages such as these, and do you see the stages happening consecutively in a more or less linear process? (If yes, go to #2; if no, go to #3)
2) If yes, does any one stage present more of a challenge than the others?
    a. What makes that stage more challenging?
3) If no, why not and how does your climate change work differ in form or process?

Thank you very much for taking the time to share this information with us. Your willingness to help with this study is greatly appreciated.
